# Air Pollution and Post-Discharge Recovery Among Older Adults Hospitalized for Heart Failure

**DOI:** 10.1093/geroni/igaf122.4320

**Published:** 2025-12-31

**Authors:** Tong Wen, Jingwen Hu, Michelle Shardell, Rozalina McCoy, Shuo Chen, Kathleen Ryan, Jason Falvey, Chixiang Chen

**Affiliations:** University of Maryland School of Medicine, Baltimore, Maryland, United States; University of Maryland School of Medicine, Baltimore, Maryland, United States; University of Maryland School of Medicine, Baltimore, Maryland, United States; University of Maryland School of Medicine, Baltimore, Maryland, United States; University of Maryland School of Medicine, Baltimore, Maryland, United States; University of Maryland School of Medicine, Baltimore, Maryland, United States; University of Maryland School of Medicine, Baltimore, Maryland, United States; University of Maryland School of Medicine, Baltimore, Maryland, United States

## Abstract

While exposure to fine particulate matter (PM2.5) has been associated with an increased risk of hospitalization for heart failure among older adults, its impact on post-discharge recovery in older adults already hospitalized for heart failure remains unclear. We evaluated associations between PM2.5 exposure during the month of hospital admission and days spent at home (DAH) as well as mortality in a nationwide representative sample of US adults aged 65 and older. Data from 66 854 Medicare Fee-for-service beneficiaries with heart failure hospitalization (2017-2019) were linked with validated, model-derived mean PM2.5 concentrations at Zip Code Tabulation Areas level. Post-discharge six-month DAH was defined as days alive minus days spent in inpatient hospitals, hospital observation units, nursing facilities, or emergency departments. All-cause mortality was assessed as time from hospital discharge to death within six-month. Quantile regression and Cox proportional regression models, adjusted for covariates, were used to quantify associations. Compared with those exposed to the lowest PM2.5 category ( < =5.9 µg/m3), those in the highest quartile PM2.5 level (>8.6 µg/m3) was associated with 4.4 fewer days (95% CI: 0.9-8.0; P = 0.014) at the 20th percentile of DAH. Exposure to the highest quartile PM2.5 level was also associated with increased six-month all-cause mortality, as compared to the lowest PM2.5 category (hazard ratio = 1.066 (95%CI: 1.022-1.113, P = 0.003). These findings suggest that air pollution may negatively impact recovery more strongly at the lower tail of recovery than at the median or higher tail, highlighting the need for targeted intervention strategies to protect the most vulnerable patients.

